# Surgery

**Published:** 1893-08

**Authors:** 


### SURGERY.

EDITED BY DR. F. W. McRAE.

### EARLY TREPHINING IN HEAD INJURIES.

Lucas Championnière (*Gaz. d. Hôp.*, June 6th) recently showed at the Société de Chirurgie a man who had been trephined three weeks before, an opening six centimeters in size being made, owing to sharp pain all over the head and an insupportable feeling of giddiness. He fell from a height of about twenty-three feet, immediately lost consciousness, and did not recover until nineteen days after, when he had forgotten all that had happened. No epileptic attacks, no paralysis. The dura mater was intact, but a grey tint of the meninges and some small white points left no doubt of the existence of commencing meningo-encephalitis. All symptoms disappeared after the trephining, as is often the case with such patients. The author lays great stress on the advantages of early interference in these cases.—*Brist. Med. Jour., June* 21.

### AN IMPROVED TECHNIQUE IN THE OPERATION OF EXTERNAL PERINEAL URETHROTOMY.

In the *New York Medical Journal*, January 21, 1893, Fluhrer describes his method of performing the operation of external perineal urethrotomy. When a filiform bougie can be introduced through the stricture he passes over it a urethrotome resembling that of Teevan, and divides all strictures anterior to the perineum. The urethrotome being withdrawn, a Gouley tunnelled staff is passed over the guide and, when the stricture is reached, it is reversed so that its break projects toward the surface of the perineum; on it a small incision is made into the urethra. A thread is then attached to each side of the urethral opening to act as retractors, and the portion of the guide in the penile urethra withdrawn through the opening. A small hook is next fitted into the extremity of the staff, and it is used to draw upward the incision in the urethra. By this means and the two threads the urethra is fully exposed. The urethrotome being threaded again on the guide is introduced into the bladder and the stricture cut on the floor. Further incision may be made on a director introduced into the bladder through the wound. If the stricture is impermeable, then endeavor to feel the urethra behind, and cut forward or open at the apex of the prostate after the method of Cock.—*University Medical Magazine.*

---

### THE HYDERABAD CHLOROFORM COMMISSIONS.

Dr. Patrick Hehir has carefully reviewed their work. The first Commission showed that the keystone to the safe administration of chloroform in man and in most of the lower animals is formed by the two principles enunciated in the following terms: (1) With regard to the heat: "The Commission consider that it is impossible for chloroform vapor to kill a dog by acting directly on the heart, and this holds good no matter in what doses or in what manner the anæsthesia is induced. Death from primary cardiac failure never took place. (2) Regarding the importance of watching the respiration: So long as the respiratory mechanism remains uninterrupted, chloroform anæsthesia is perfectly safe, but should the respiration be impeded and the elimination of chloroform be thereby interfered with, danger at once arises; in other words, if respiration be kept regular, no danger can arise." The essential object in view was to show that the principles upon which Syme administered chloroform were sound and reliable. Free admixture of air with the vapor of chloroform, which is insured by using some soft, porous material (as a towel or lint) presenting a large surface; no stint in the quantity of chloroform given; and watching the respiration. He considered that there was never any occasion to feel the pulse or examine the heart, either before or during anæsthesia. The second Commission brought out the practical points that (*a*) primary cardiac syncope during chloroformization does not occur; and (*b*) the maintenance of regularity of the respiration is absolutely indispensable to the safe administration of chloroform. By keeping the respiration regular the force of blood-pressure is kept regular also, as is likewise the action of the heart and the circulation generally. Under these circumstances it would be impossible to administer chloroform in poisonous doses unless it were continued after the occurrence of complete anæsthesia. A grand rule to follow in practice is to remove the chloroform and allow the patient to breathe fresh air for a second or two when at all in doubt. —*Medical Chronicle*, and *Indian Medical Gazette*.

---

### THE VALUE OF INTERNAL MEDICATION IN THE TREATMENT OF MALIGNANT DISEASE.

In discussing the treatment of malignant disease by internal medication, Wright (*Annals of Surgery*, April, 1893) does not uphold this method to the exclusion of surgical interference, but rather advises its use in preparation for and in conjunction with the most thorough extirpation by the knife. For the purpose of destroying outlying foci of disease left by the knife, he tried various drugs internally, and in cases that he had operated upon as well as in those in which operation was impossible, he began to give the bromide of arsenic in one-fortieth to one-tenth grain doses after meals, and the carbonate of lime before meals in five to ten grain doses in the tincture of calumba. In many cases coming under the head of sarcoma, there was quite a rapid tendency toward cure, and this was generally permanent. Large deposits, as a rule, would not yield, but excision of the enlargement was often followed by a sure cure. As to cases affecting bone, osteo-sarcoma, the treatment was not so favorable, but seemed of some value. He advises practitioners to give the bromide of arsenic to all patients as soon as they come under their care, and continue the remedy for a long time after operation. In a considerable number of cases operated upon for cancer in the past three, four and five years, to whom the bromide of arsenic was administered for a time, say six or twelve months, complete health has been restored and the scar tissue is now in every way just as normal as it would be if the wound had been in perfectly healthy tissue. In no case did the microscopic examination fail to confirm the diagnosis. The same results might possibly have been obtained by complete and thorough operation, and yet he believes there is value in the after-treatment, and that a case would be neglected without it. In inoperable cases he has seen good results, not the removal of the growth, but relief of pain and retardation of the growth. In some cases of cancer of the intestines he has seen the bromide of arsenic bring relief and prolongation of life.—*Journal of American Medical Sciences.*

---

### DRAINAGE OF THE URETERS AFTER HYPOGASTRIC OPERATIONS UPON THE BLADDER.

Willems (*Annales de la Society de Medicine de Gand*, 1892) advocates the practice, which, he states, is a delicate yet perfectly practicable proceeding. In hypogastric lithotomy it is advisable whenever the wound in the bladder is unimportant and subject to prejudicial influences, when the urine is unhealthy and septic. The ureters should also be drained after the removal of a vesical tumor, whether by hypogastric or perineal incision. It protects the wound from morbid urine, and allows of antiseptic plugging of the cavity. He places the patient in the Trendelenburg position, the pelvis being raised about fifteen inches off the table. The abdominal viscera no longer press the bladder walls together, and the trigone can be readily seen through the hypogastric incision. A catheter with a Y-shaped end is then passed backward through the urethra. The two branches are next introduced through the ureter. The upper part of the mucous fold over the orifice of the ureter should be seized with forceps of the kind used for fixing the conjunctiva, otherwise the very movable vesical mucous membrane may be pushed forward. As the vesical part of the ureter is a narrow tunnel, the passage of the catheter needs skillful manipulation. The ureter tolerates the catheter very fairly. In a case of Paulik's the woman's bore it for forty-eight hours, in Schede's case seven days, and in Albarran's case ten days. Hence the catheter allows time for perfect union of the vesical wound.—*Universal Medical Magazine, June,* 1893.

